# Commentary: Risk factors for mild cognitive impairment in type 2 diabetes: a systematic review and meta-analysis

**DOI:** 10.3389/fendo.2025.1681637

**Published:** 2025-11-05

**Authors:** Shanshan Wu

**Affiliations:** Clinical Laboratory Department, The Affiliated Hospital of Liaoning University of Traditional Chinese Medicine, Shenyang, Liaoning, China

**Keywords:** type 2 diabetes mellitus, diabetes mellitus, mild cognitive impairment (MCI), risk factors, meta-analysis, sex

## Introduction

We read with great interest the article by Zhao and colleagues entitled “Risk factors for mild cognitive impairment in type 2 diabetes: a systematic review and meta-analysis” (1). We acknowledge Zhao et al.’s systematic review elucidating risk factors for mild cognitive impairment (MCI) in type 2 diabetes (T2DM) (1). Their identification of advanced age (≥60 years), prolonged diabetes duration (8–9 years), elevated HbA1c (>9%), low education (≤6 years), and cardiometabolic comorbidities as significant MCI predictors advances this field. Furthermore, smoking, hypertension, cardiovascular disease (CVD), insulin resistance, fasting plasma glucose (FPG), and high-sensitivity C-reactive protein (HS-CRP) were also significantly linked to higher MCI risk.

However, methodological concerns regarding subgroup analyses warrant critical examination. A discrepancy exists between the statistical protocol (Section 2.5) and its implementation in Figure 8A. Per protocol specifications, a random-effects model is mandated when heterogeneity exceeds 50% (*I*² ≥ 50%). Given the ongoing controversy regarding sex differences in the risk of MCI among T2DM patients, Zhao et al. performed subgroup analyses by sex. The authors state in their original article (page 8, line 23) that a random-effects model was used due to significant between-group heterogeneity in effect sizes (*I*² = 81.5%; Figure 8A). However, Figure 8A reveals that a fixed-effects model was erroneously applied for this analysis. This discrepancy potentially biases the sex-specific risk estimates, leading to an erroneous conclusion that female sex is an independent risk factor for MCI in individuals with T2DM (1). This commentary aims to rectify the erroneously applied models mentioned above and to provide a correct reanalysis.

## Statistical analysis

We strictly adhered to the same dataset and inclusion criteria as those described by Zhao et al. Statistical analyses were conducted utilizing the RevMan software (version 5.3). Dichotomous outcome variables were evaluated using odds ratios (ORs) accompanied by 95% confidence intervals (CIs). Heterogeneity among the included studies was quantified employing the *I*² statistic, with conventional thresholds designating low, moderate, and high heterogeneity as *I*² values exceeding 25%, 50%, and 75%, respectively (2). The fixed-effects model, which presumes homogeneity of effect sizes across studies, was applied in instances of low heterogeneity (*I*² ≤ 50%); this model derives the pooled effect estimate via inverse-variance weighting. Conversely, the random-effects model, accommodating inherent variability in effect sizes, was implemented where significant heterogeneity was present (*I*² > 50%). This approach incorporates between-study variance through inverse-variance weighting. Consequently, model selection was determined *a priori* based on the observed *I*² value: the random-effects model was employed when *I*² exceeded 50%; otherwise, the fixed-effects model was utilized.

## Revised meta-analysis results

**Figure 1:**
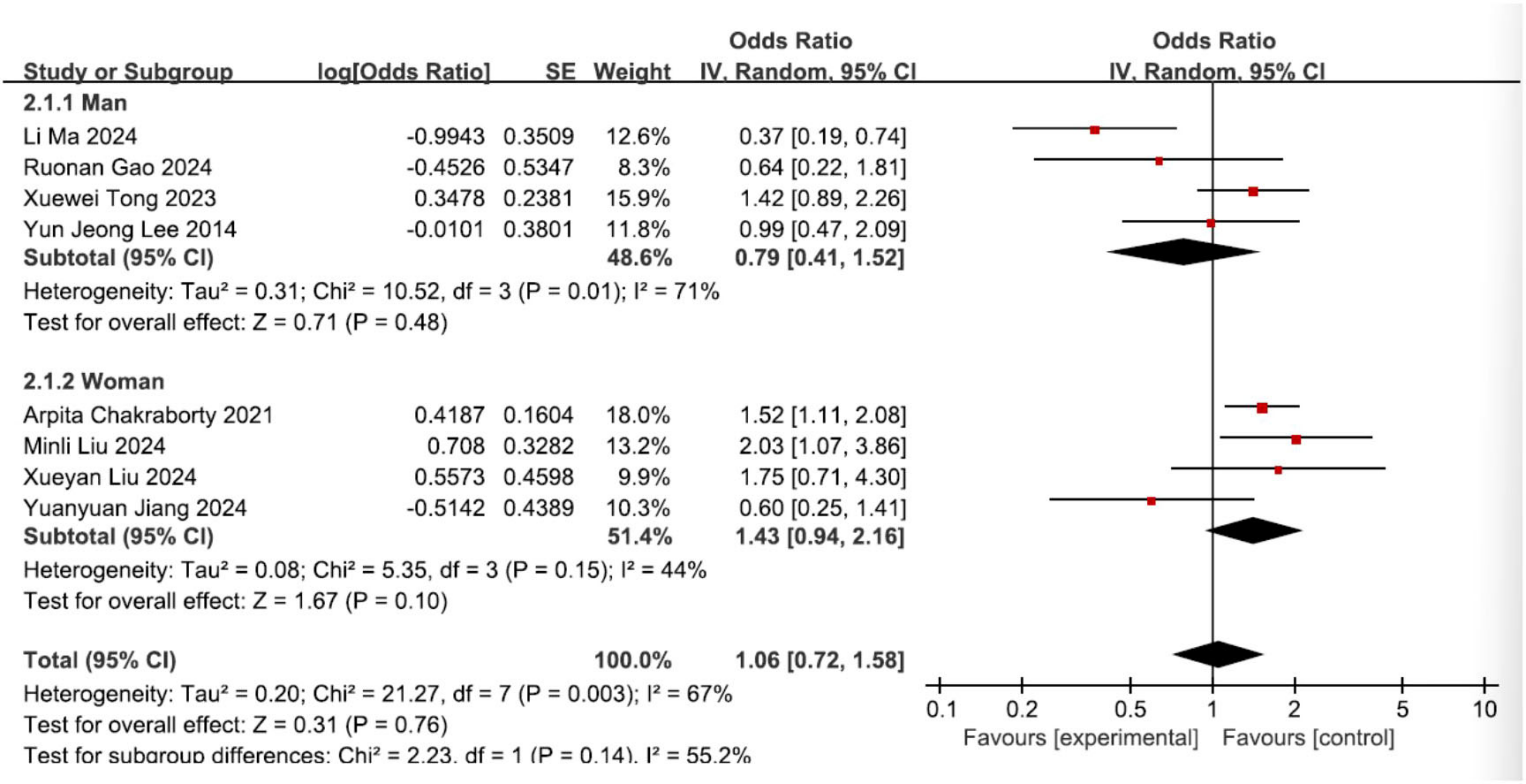
Forest plot of sex.

A reanalysis of the sex-stratified data (from eight studies) demonstrated significant between-group heterogeneity in effect sizes *(I*² = 55.2%; **Figure 1**); therefore, a random-effects model was employed. The results indicate that female gender is not an independent risk factor for MCI in individuals with T2DM (OR = 1.43, 95% CI: 0.94–2.16, *P* = 0.10; **Figure 1**), contrasting with Zhao et al.’s conclusion of equivalent predictive power to age, HbA1c, and education. Analysis indicated that male sex was not an independent risk factor for MCI development in patients with T2DM (OR = 0.79, 95% CI: 0.41–1.52, *P* = 0.48; **Figure 1**), consistent with the findings reported by Zhao et al.

## Discussion

The association between MCI and female sex in patients with T2DM remains a subject of considerable controversy in current literature. The controversy surrounding sex-specific MCI risk in T2DM likely originates from the following:

1. Socio-confounders: Women may exhibit elevated apparent risk due to fewer educational opportunities, lower adherence to health management, and higher prevalence of cardiovascular comorbidities (3).
2. Divergent biological mechanisms: Neuroprotective effects of estrogen become attenuated in postmenopausal women (4), while men may experience earlier onset of insulin resistance-associated brain injury (5).
3. Metric heterogeneity: Sex-dimorphic biomarkers [e.g., body roundness index with female-predominant predictive accuracy (4)] are considered.

For instance, Liu et al. identified female gender as a factor significantly associated with cognitive impairment in patients with T2DM (3). Similarly, Yanting Liu et al. identified the body roundness index (BRI) as a robust predictor of cognitive impairment in elderly individuals with T2DM, demonstrating significantly higher predictive accuracy in women compared to men (6). A recent study by Chu et al. demonstrates that an elevated uric acid to high-density lipoprotein cholesterol ratio (UA/HDL-c) is associated not only with overall cognitive function in female T2DM patients but also with specific impairments in executive function and visuospatial abilities. However, this association was not observed in male patients (7). A body of evidence indicates that sex hormones modulate uric acid and triglyceride metabolism (8–12), which may explain the sex-based variations in the UA/HDL-c ratio. Furthermore, separate research links sex hormones to the regulation of cognitive function (13, 14), offering a potential explanation for the observed sex differences in cognitive outcomes. The current body of evidence exhibits limitations. Consequently, future study designs should incorporate sex-specific assessment of MCI risk in T2DM to reduce potential confounding effects on research outcomes.

In conclusion, while Zhao et al. report female sex as a significant MCI predictor in T2DM, reanalysis using appropriate random-effects models negates this association. Future meta-analyses require stringent methodological rigor to resolve sex-specific risk controversies influenced by socio-biological confounders. We trust that these methodological considerations will enhance the rigor and validity of the published findings. We appreciate the opportunity to contribute to the scientific discourse on this critical issue and anticipate a constructive resolution of the raised concerns.

## References

[1] Zhao Y Wang H Tang G Wang L Tian X Li R. Risk factors for mild cognitive impairment in type 2 diabetes: a systematic review and meta-analysis. Front Endocrinol. (2025) 16:1617248. doi: 10.3389/fendo.2025.1617248, PMID: 40589509 PMC12206637

[2] Higgins JPT Green S. Cochrane handbook for systematic reviews of interventions version5.1.0 updated March 2011. Hoboken A John Wiley & Sons, Ltd., Publication. (2011).

[3] Liu M Wang Z Han J Mu Z Bian H. Analysis of current situation and influencing factors of cognitive dysfunction associated with type 2 diabetes and follow-up study on treatment effectiveness. Front Neurol. (2024) 15:1664–2295. doi: 10.3389/fneur.2024.1419017, PMID: 39220736 PMC11362961

[4] Zhang Y Tan X Tang C. Estrogen-immuno-neuromodulation disorders in menopausal depression. J Neuroinffamm. (2024) 21:(1742–2094 (Electronic)). doi: 10.1186/s12974-024-03152-1, PMID: 38898454 PMC11188190

[5] Luders E Poromaa IS Kurth F. The neuroanatomy of menopause. Hormones Behav. (2025) 172:1095–6867. doi: 10.1016/j.yhbeh.2025.105749, PMID: 40334636

[6] Liu Y Qiu H Zhang M Lin J. Sex-specific association between body roundness index and cognitive impairment among hospitalized middle-aged and elderly patients with type 2 diabetes in China: a cross-sectional analysis. Eur J Med Res. (2025) 30:2047–783X. doi: 10.1186/s40001-025-02849-0, PMID: 40615897 PMC12231750

[7] Chu Y Wang L Guo Q Chang Y Lu N Gao Q. Sex differences in the association between the uric acid to high density lipoprotein cholesterol ratio and mild cognitive impairment in patients with type 2 diabetes mellitus. Front Nutr. (2025) 12:1667948. doi: 10.3389/fnut.2025.1667948 41178941 PMC12577417

[8] Guo A Chen P Cao J Wu C Ding S. Association between sex steroid hormones and α-klotho: Results from the NHANES 2013–2016 and Mendelian randomization study. Exp Gerontol. (2025) 201:1873–6815. doi: 10.1016/j.exger.2025.112699, PMID: 39900258

[9] Chen H Feng WD Feng JL Zhao C Gao ZX Wang B. Association of serum uric acid with male sexual hormones and erectile dysfunction: a bidirectional 2-sample Mendelian randomization analysis. Sex Med. (2024) 12:2050–1161. doi: 10.1093/sexmed/qfae051, PMID: 39156235 PMC11330324

[10] Liu YF Wang HH Geng YH Han L Tu SH Chen JS. Uncovering the potential mechanisms and effects of hyperuricemia and its associated diseases on male reproduction. Reprod Sci. (2024) 31:2184–98. doi: 10.1007/s43032-024-01453-7, PMID: 38379071

[11] Zhao H Wang D Xing C Lv B Wang X He B. Pioglitazone can improve liver sex hormone-binding globulin levels and lipid metabolism in polycystic ovary syndrome by regulating hepatocyte nuclear factor-4α. J Steroid Biochem Mol Biol. (2023) 229:1879–220. doi: 10.1016/j.jsbmb.2023.106265, PMID: 36737028

[12] Ha LX Li WX Du YD Yuan YY Qu XX. Tumor necrosis factor alpha level in the uterine fluid of patients with polycystic ovary syndrome and its correlation with clinical parameters. J Inflammation Res. (2022) 15:1178–7031. doi: 10.2147/JIR.S382808, PMID: 36339827 PMC9628701

[13] Dratva MA-O Banks SJ Panizzon MS Galasko D Sundermann EE. Low testosterone levels relate to poorer cognitive function in women in an APOE-ϵ4-dependant manner. Biol Sex Differ. (2024) 15:2042–6410. doi: 10.1186/s13293-024-00620-4, PMID: 38835072 PMC11151480

[14] Chang YT Chen YA-O Kang HA-O. Revealing the influences of sex hormones and sex differences in atrial fibrillation and vascular cognitive impairment. Int J Mol Sci. (2021) 22:1422–0067. doi: 10.3390/ijms22168776, PMID: 34445515 PMC8396287

